# Tumor Heterogeneity, Single-Cell Sequencing, and Drug Resistance

**DOI:** 10.3390/ph9020033

**Published:** 2016-06-16

**Authors:** Felix Schmidt, Thomas Efferth

**Affiliations:** Department of Pharmaceutical Biology, Institute of Pharmacy and Biochemistry, Johannes Gutenberg University, Staudinger Weg 5, 55128 Mainz, Germany; felixschmidt.online@googlemail.com

**Keywords:** intratumoral heterogeneity, tumor ecosystems, single-cell sequencing, micromanipulation, laser-capture microdissection, flow cytometry, next generation sequencing, RNA sequencing, whole genome amplification, multi-region sequencing, circulating tumor cells, xenograft tumor models, cancer treatment, individualized therapy, precision medicine

## Abstract

Tumor heterogeneity has been compared with Darwinian evolution and survival of the fittest. The evolutionary ecosystem of tumors consisting of heterogeneous tumor cell populations represents a considerable challenge to tumor therapy, since all genetically and phenotypically different subpopulations have to be efficiently killed by therapy. Otherwise, even small surviving subpopulations may cause repopulation and refractory tumors. Single-cell sequencing allows for a better understanding of the genomic principles of tumor heterogeneity and represents the basis for more successful tumor treatments. The isolation and sequencing of single tumor cells still represents a considerable technical challenge and consists of three major steps: (1) single cell isolation (e.g., by laser-capture microdissection), fluorescence-activated cell sorting, micromanipulation, whole genome amplification (e.g., with the help of Phi29 DNA polymerase), and transcriptome-wide next generation sequencing technologies (e.g., 454 pyrosequencing, Illumina sequencing, and other systems). Data demonstrating the feasibility of single-cell sequencing for monitoring the emergence of drug-resistant cell clones in patient samples are discussed herein. It is envisioned that single-cell sequencing will be a valuable asset to assist the design of regimens for personalized tumor therapies based on tumor subpopulation-specific genetic alterations in individual patients.

## 1. Intratumoral Heterogeneity

Intratumoral heterogeneity (ITH) has been well-known for six decades, as scientists initially put forward the concept that a single tumor consists of many cell subpopulations. As pointed out by Peter Nowell, tumor evolution and microenvironmental influences on neoplastic cells are causes for rising mutability in tumor cells [1]. Reports on the morphological heterogeneity in colorectal carcinomas trace back to the 1950s [2]. Gloria Heppner advanced a definition of the term tumor heterogeneity that is still valid today [3]. A new perspective on this topic has been recently published [4,5]. The authors stated that cancer is “a metaphor of organismal life, for Darwin evolution in real time” and posed the question, “what if cancer is not a metaphor, but rather an example of life itself?”

In the view of Charles Darwin, the fittest organism with the best adaptation is best suited to survive. Allison and Sledge projected this to cancer and discovered cancer as an ecosystem. At this point, it is particularly important to highlight that tumors can better adapt and survive with increasing malignancy. This means that malignant cells can much easier and faster generate highly heterogeneous descendants. The many different subclones can then build a strong society of cells and form a broad-based heterogeneous tumor.

Another important point brought up by Heppner and Shekhar was the societal relationship between cancer subpopulations, which enables reciprocal influences in growth rate, metastasis, immune sensitivity, and chemotherapeutic response [4].

The concept of tumor heterogeneity has a major impact on therapeutic approaches. Cancer therapy needs to become more personalized, selective, and specific. The biggest challenge here is to capture all subpopulations of a tumor. All different genomic subclones must be detected to kill all tumor cell populations. This represents a tremendous technical challenge, as pointed out below. If not efficiently captured, drug-resistant subpopulations will emerge. According to Darwin’s diction, it can be postulated that the fittest population will repopulate the territories of cell populations killed by chemotherapy [3].

In order to achieve a therapeutic effect, it would be useful to involve the social characteristics of cancer [4]. From an evolutionary point of view, it is much easier for heterogeneic malignancies to disperse, because the therapeutic elimination of a constantly changing population can hardly be reached. This postulation implies a change in current therapeutic strategies.

## 2. Single-Cell Sequencing

To better understand ITH, it is necessary to sequence the diverse genomes of tumor cell populations. Nicholas Navin is called the father of single-cell sequencing [6]. His studies reinforced the concept that the complexity of ITH can be addressed by only a single tumor cell genome.

It represents a considerable technical challenge to isolate and sequence a single cell. As Navin said: “Cells like to stick together” [7]. It is difficult to get the nucleus of only one cell, because neighboring nuclei could be hurt and adulterate the result during isolation. In addition, the question arises of how to sequence the sparse amounts of DNA from a single cell. The time was ripe because novel and innovative technologies recently emerged, enabling the management of these tasks.

**Figure 1:**
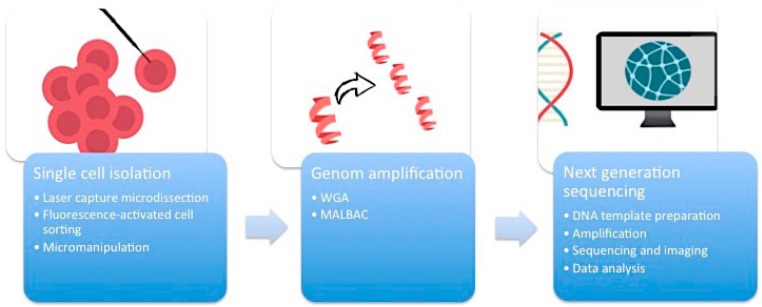
The work-flow of single-cell sequencing.

First of all, it is essential to manage the isolation of one single cell from the complex heterogeneous tumor ecosystem from tissue samples or cell cultures (Figure 1). There are several methods available to accomplish this task.

### 2.1. Micromanipulation

Micromanipulation represents an opportunity to isolate single cells from cell cultures or liquid samples such as sperm, saliva, or blood. This method is based on the mechanical shearing of cells. Complex micromanipulation systems translate intricate movements (in small micrometer dimensions) into fine and harmonious motions of instruments. This method is time- and labor-intensive.

### 2.2. Laser-Capture Microdissection (LCM)

Laser-capture microdissection (LCM) was developed by Emmert-Buck and colleagues [8]. This system allows for the direct isolation of single cells from heterogeneous human tissue sections. A transparent thermoplastic film (ethylene vinyl acetate polymer) is applied to the surface of the tissue section on a standard glass histopathology slide. A carbon dioxide laser pulse then specifically activates the film above the cells of interest. Strong focal adhesion allows for the selective procurement of targeted cells. The advantages of LCM include the clinical applicability and the possibility of labeling specific cells with fluorescent or chromogenic antibodies. A disadvantage is that some nuclei can be hurt during tissue sectioning, which will distort the accuracy of the data. One advancement is expression microdissection, which is a real high-throughput isolation tool [9].

### 2.3. Flow Cytometry Using Fluorescence-Activated Cell Sorting (FACS)

Flow cytometry using fluorescence-activated cell sorting (FACS) is the most efficient method of isolating a large number of single cells or nuclei from liquid suspensions [10]. Flow cytometry is based on the emission of optical signals from a cell, if it passes a laser beam. The probe reaches a quartz or glass micro-channel, which is so thin that only one cell can pass the laser beam at a time, allowing for the recording of a signal. FACS has the additional advantage that cells can be labeled with fluorescent antibodies or nuclear stains (4′,6-diamidino-2-phenyl indol dihydrochloride, DAPI) and sorted into different fractions for downstream analysis [6].

### 2.4. Whole Genome Amplification (WGA)

One cell yields DNA in the nanogram range, which is not enough for DNA sequencing. Therefore, a robust DNA polymerase is needed, which displays sufficient processivity for DNA synthesis in microgram quantities. Phi29 DNA polymerase from *Bacillus subtilis* fulfills this requirement [11]. Moreover, the *Bacillus stearothermophilus* polymerase can be used for robust and reliable human genome amplification. This process is called multiple displacement amplification (MDA) [12]. An alternative method of WGA is called multiple annealing and looping-based amplification cycles (MALBAC). This technique combines advantages of MDA and improved PCR. Random primers with a 27-nucleotide sequence, specific DNA polymerases, and a simple 18-cycle PCR run is enough to get a representative amount of DNA with less amplification bias and higher levels of specificity and reproducibility than MDA [13,14].

The breakthrough of next-generation sequencing (NGS) or RNA-sequencing (RNA-Seq) has fundamentally changed the perspectives on genome profiling. This technology allows for the rapid and reliable detection of all 3.2 billion base pairs of the human genome. Massive parallel sequencing processes produce roughly 1 GB of data of DNA information per single run. This is the main technical milestone that makes single-cell sequencing indispensable for research on ITH. The core of single-cell sequencing is based on three different processes. Specific innovative techniques are available for each of these steps.

The Roche/454 FLX Pyrosequencer was introduced in 2004. The 454 sequencing technology uses emulsion PCR (emPCR) for the clonal amplification of probes, followed by high parallel pyrosequencing. With emPCR, the DNA is bound to specific beads, which are located together with desoxyribonucleotides in emulsion drops. Through this process, the DNA is clonally amplified. Furthermore, these DNA-coupled beads are enriched and loaded into individual PicoTiterPlate (PTP) wells. The PTP wells contain all sequencing reagents. Thereafter, wells are incubated with either dATP, dTTP, dCTP, or dGTP. Each incorporation of a desoxyribonucleotide by DNA polymerase results in the release of a pyrophosphate, which initiates light production by sulphurylase and firefly luciferase, where the amount of light is proportional to the number of incorporated nucleotides [15]. Sulphurylase catalyzes the reaction of pyrophosphate to ATP, which is used by luciferase to catalyze luciferin to oxyluciferin. The latter reaction is accompanied by the production of light. The light signals can be assigned to the specific dNTP leading to the assembly of the DNA sequence.

The Illumina Genome Analyser was introduced in 2005. The main characteristic of this system is the specific adapter library on an oligo-derivatized flow cell surface. For template preparation, the bridge amplification technique is used to amplify the clonal DNA strand, which generates higher signal intensities and therefore better statistical results. Forward and reverse primers, which are covalently attached to a flow cell surface, are extended by a polymerase and can bridge to complementary oligos. Millions of DNA fragments are amplified by repeated denaturation and extension steps. The generating free ends of the DNA fragments then hybridize to sequencing primers to start sequencing reactions. Illumina also uses 3′-blocked nucleotides. The nucleotides are labeled with fluorophores and blocked at the 3′-OH end in a reversible manner. Because of the fluorescent modified nucleotides, the imaging process and data analysis is based on fluorescent emission detection.

Other NGS systems are the SOLiD™ System (a two-base encoding technology, which provides inherent error correction), the Helicos Heliscope™, and the SMRT System by Pacific Biosciences [15,16]. Because of the sequencing quality control (SEQC) in 2014, it is easier now to distinguish between NGS systems [17].

## 3. Multi-Regional Sequencing Studies

The next step is to apply NGS technologies in a clinical setting to take considerable advantage of the full potential to understand the complexity of ITH. Some landmark awareness came from the use of NGS for multi-region sequencing of lung adenocarcinoma samples. Evidence was gained for regional ITH based on single tumor biopsies to portray the mutational landscape. ITH patterns may be different between cancer types. Multiregion sampling is required to assess the full ITH complexity of gene mutations in tumors. Further studies are needed to investigate biopsies at diagnosis and at relapse to fully understand the clinical impact of ITH [18].

A multiregional sequencing study of primary breast cancer was recently published. The authors found interesting causalities between different subclones in heterogeneous tumor tissues by using Illumina HiSeq^®^ for genomic sequencing. They discovered correlations between the tumor size and the degree of heterogeneity in triple negative breast cancer. Probably, tumors with profound heterogeneity grow to larger sizes [19].

NGS paved the way for new approaches to model the evolution of individual cancers. The power of these models is increased if tumor samples from multiple sites are sequenced. Other authors have provided bioinformatics tools to construct phylogenetic trees based on complex gene mutation patterns of tumors [20].

Another recent pilot study addresses the question of whether it is possible to detect ubiquitous and heterogeneous somatic mutations in cell-free DNA (cfDNA) isolated from blood plasma obtained prior to surgical resection of the tumor. For that purpose, biopsies of stage I and II primary non small-cell lung cancer tumors (NSCLC) were used for a multi-region whole exome sequencing and validation by AmpliSeq to explore genetic changes such as gene fusions, single nucleotide polymorphisms, and copy number variations. Selected genetic alterations found in cfDNA were searched in tumor DNA by using multiplex PCR coupled with NGS. This mPCR-NGS approach revealed considerable ITH in NSCLC. With more advanced techniques suited to use cfDA for routine diagnosis, novel biomarkers in blood plasma may be developed to monitor clonal dynamics of tumors in clinical practice [21].

## 4. Multifactorial Drug Resistance

Drug resistance represents an everlasting obstacle in cancer treatment and is always linked to tumor progression and worse prognosis. Resistance to chemotherapeutic agents can be divided into two types: intrinsic or acquired resistance. Intrinsic resistance is present prior to chemotherapy exposure, and the tumor fails to respond to initial treatment. Acquired resistance occurs only during or after the course of treatment [22]. In general, two factors are responsible for drug resistance: (1) individual specificity, where poor absorption of orally administered drugs increased drug metabolism or increased excretion limit drug delivery to the tumor masses [23,24]; (2) cancer cell specificity, where genetic and epigenetic alterations affect drug sensitivity.

Clinical drug resistance is characterized by resistance not only to one or a few drugs, but frequently to a broad spectrum of drugs, even if these drugs have never been applied before to a specific patient [25]. In the past several years, this phenomenon has been investigated, and the multidrug resistance (MDR) phenotype has been described as the underlying mechanism. MDR is defined as the cross-resistance of cancer cells to a wide variety of anticancer drugs, which are structurally or functionally unrelated [26].

Several cellular mechanisms that enhance the occurrence of MDR have been identified, such as (1) increased drug efflux as a result of overexpression of ATP-dependent efflux pumps; (2) activation of detoxifying systems such as cytochrome P450 and glutathione *S*-transferase; (3) decreased drug uptake; (4) mutated oncogenes and tumor suppressor genes; (5) activation of DNA repair capacity, (6) activation of pro-survival signaling pathways; and (7) inactivation of apoptosis pathways [27,28,29]. To this end, in any population of cancer cells that is exposed to chemotherapy, more than one mechanism of MDR can be present [30] and these resistance mechanisms worsen survival chances of cancer patients [31]. Hence, treatment strategies have to be developed fighting resistant tumors with multiple resistance mechanisms [32,33,34,35]. On the other hand, it is very common that cancer cells are genetically heterogeneous. The expression of these resistance mechanisms is not uniform in all cells of a tumor, and several different mechanisms are operative at the same time [25,36].

## 5. Circulating Tumor Cells

The problem of heterogeneity applies not only to MDR tumors, but to tumors resistant to any drug. The sequencing of single cells will facilitate the detection of even the smallest populations of resistant cells. A proof-of-principle of this concept has recently been published [37]. The authors collected 77 circulating tumor cells of 13 prostate cancer patients. While the sampling of metastasizing cells in affected organs remains difficult, they can be isolated from blood, since circulating tumor cells (CTCs) enter peripheral blood and seed metastases [18]. Collecting CTCs from blood has also been termed “liquid tumor biopsy.” Miyamoto and colleagues found considerable heterogeneity, including expression of androgen receptor (AR) gene mutations and splicing variants in the circulating prostate tumor cells by RNA sequencing [37]. Retrospective analyses of CTCs from patients progressing under treatment with an AR inhibitor, revealed a statistically significant activation of non-canonical Wnt signaling compared with untreated cases. This can be taken as evidence that single-cell analyses of CTCs do not only demonstrate genetic tumor heterogeneity. It also shows that heterogeneity contributes to treatment failure.

By using WGA and sequencing, Jiang and colleagues demonstrated that 29% of somatic single nucleotide variations (SSNVs) of a patients advanced prostate cancer tissue were also identified in isolated CTCs. Furthermore, 86% of clonal mutations in CTCs could be traced back to primary or metastatic tumors [38]. Hence, analysis of CTCs represents a novel and attractive approach to investigate molecular heterogeneity and may allow real-time monitoring of cancer biology in individual patients [39].

Another interesting aspect is the aberrant expression of stroma-derived extracellular matrix (ECM) proteins in CTCs. The knockdown of these proteins inhibited cell migration, invasiveness, and metastatic risk [40]. The genes in CTCs expressing ECM proteins may serve as new targets for cancer therapy. The absence of microenvironmental communication mediated by ECM proteins suppresses the spread of cancer to distant organs. In addition, the cell junction component plakoglobin is abundant in single CTCs as identified by single-cell resolution RNA sequencing. The knockdown of plakoglobin *in vivo* prevented lung metastases. The suppression of plakoglobin-dependent intercellular adhesion may stop the metastatic spread of breast cancer in patients [41].

CTCs are extremely rare in blood, and the molecular characterization of CTCs is difficult, because their isolation still represents a major technological challenge [42]. Nevertheless, by using reliable systems to separate and analyze the molecular characteristics of CTCs, e.g., by the combination of CellSearch^®^ and DEPArray™, the recovery of single CTCs can be achieved [42]. The CellSearch^®^ system is based on ferrofluid- and fluorochrome-coupled antibodies with high binding affinities for the EpCAM antigen of CTCs. After immunomagnetic capture and enrichment, fluorescent reagents are added for the identification and counting of CTCs. The DEPArray™ technology enables the manipulation and collection of cells. A circuitry with individually controllable electrodes creates a dielectrophoretic (DEP) cage around cells. This allows for the moving of single cells to specific locations on the cartridge for cell–cell interaction studies or isolation. Afterwards, WGA and sequencing can be performed [39,43].

## 6. Xenograft Tumors as Experimental Models

The methodology to transplant human tumors into mice traces back to the first half of the 20th century [44]. Today, this xenograft technology still belongs to the standard repertoire in cancer research. Complex mutation profiles of xenograft tumors have been analyzed by single-cell RNA sequencing.

In lung andenocarcinoma xenograft tumor cells, specific single nucleotide variations have been found, including KRAS (G12D). Cells with this mutation survived *in vitro* better than others [45]. These results open the possibility to develop experimental models to predict treatment outcome in heterogeneous tumors.

Furthermore, clone-specific population dynamics have been monitored in breast cancer xenografts and their corresponding metastases [46]. Subclonal phylogenetical investigations of multiple myeloma xenograft tumors revealed the parallel evolution of two independent clones, which both had an activated RAS/MAPK pathways by *RAS* mutation. Clonal diversity and selective pressure represent important reasons for tumor progression and treatment resistance in myeloma [47].

## 7. Precision Medicine

It has always been known that every patient’s tumor is unique and therefore requires individual treatment for optimal therapy outcome. Hence, tailored treatments based on individual genetic profiles may be more promising to sustainably fight cancer than conventional standard chemotherapy. “What if matching a cancer cure to our genetic code was just as easy, just as standard?” as US President Barack Obama recently stated [48].

Precision medicine as a new emerging therapeutic strategy to improve treatment success of patients has gained enormous attention from both health professionals and the general public [49]. The main principle of precision medicine in clinical oncology is to treat patients on the basis of their individual genetic mutations [50]. Single-cell sequencing may help to realize the dream of precision medicine in cancer pharmacology.

A recently published approach of individual therapy in a multi-dimensional clinical genomics study of children and adolescent young adults with relapsed non-central nervous system solid cancers may provide a roadmap for integrative genomic analyses, robust bioinformatics, and common threads for future precision therapy protocols [51].

As outlined by Stephen *et al.* [52], several conditions determine the development of patient-tailored treatment protocols, e.g., (1) comprising meta-analyses on DNA sequencing results and related implications for drug development, and (2) the availability of individual targeted agents and biomarkers for therapy monitoring (e.g., liquid biopsy). The future of clinical precision medicine lies before us, but we proceed with big steps.

## 8. Limitation of Single Cell Sequencing

Besides all enthusiasm, single cell sequencing has some limitations. The inadvertent risks are not only higher error rates and misreads or mismatches in base pairs. Current genome sequencing still requires too much time, personal effort, and money. At this point, it is important to mention that the costs have significantly dropped down over the past decade [53]. Data from the National Human Genome Research Institute (NIH) shows a reduction from $10 million (2006) to only about $1000 (2016) per genome [54]. Even $1000 is still too much because of the annually high cancer incidence (about $14 million in 2012). Another challenge is the deficit of standardized quality control metrics and the still insufficient number of PhD students and scientists with a background in bioinformatics. To traverse deeper into the technical considerations, the single-cell sequencing process can be divided in three parts: (1) data generation, (2) data analysis, and (3) data sharing and storage:

(1) To generate data, lengthy approval processes and laborious requirements cannot keep pace with technological improvements. A key to solving this problem might be cooperative research agreements for operation and maintenance funds or the employment of experienced personnel who work closely with contracting agencies. Furthermore, the number of full-time staff is limited. More advertising for molecular laboratory technicians and PhD students in molecular and bioinformatic genetics is needed. Long-read single-molecule platforms, such as PacBio RS II and Sequel sequencers, should be available. The development of smaller and less expensive gadgets is another requirement if sequencing should enter routine diagnostics. Compared with research laboratories, clinical routine laboratories rarely have staff skilled enough to prepare high-quality DNA libraries [55]. Educational and training enterprises or automatic library preparation may be solutions to this problem [56].
(2) The limited access to open source and analytical software impedes data analysis. At this point, more networking among basic scientists and clinicians would be desirable.
(3) The sequencing of large volumes create extraordinary burdens in terms of the sharing and storage of data. Possible mitigation includes the use of FASTA/FASTQ to email sequences and cloud-based solutions [55].

Finally, a limitation of utmost relevance are the reading errors of sequencing platforms. A trustworthy sequence quality is reached in the Human Genome Project (HGP) with error rates of less than 1 in 100,000 base pairs [57]. However, the completion of HGP took 13 years (1990–2003). New technical innovations in genome sequencing in the future will not only speed up sequencing times but also decrease error rates [16].

## 9. Conclusions

Without a doubt, we are at the dawn of the era of genome medicine. The complexity of genetic heterogeneity in tumor evolution represents a considerable scientific challenge that can only be managed by the integration of elaborated techniques of single-cell sequencing, bioinformatics, and individualized drug development. On the way to reach the ultimate goal of precision medicine, *i.e.*, the delivery of effective individual drugs for each individual patient, a considerable amount of basic research needs to be done to understand the fundamentals of tumor heterogeneity. In this respect, the use of suitable experimental models (e.g., cell lines and xenograft tumors) and patient material (e.g., solid tumor tissues and liquid biopsies) will be complemented by technical advancements that allow rapid and affordable DNA sequencing methodologies for routine diagnostics in the clinic. While it can be imagined that this approach will succeed in the years to come, an even bigger challenge may be the development of large batteries of cancer drugs forgetting specific cancer mutations.

Technological developments in pharmaceutical research (e.g., high throughput screening and combinatorial chemistry) and the clinical approval of targeted drugs must keep pace with genomic progresses to fulfill the promises of precision medicine in cancer therapy.

## Abbreviations

cf DNA	circulating free DNA
CTC	circulating tumor cell
ECM	extracellular matrix
emPCR	emulsion polymerase chain reaction
FACS	flow-activated cell sorting
ITH	intratumoral heterogeneity
LCM	laser capture microdissection
MLBAC	multiple annealing and looping-based amplification cycles
MDA	multiple displacement amplification
MDR	multidrug resistance
mPCR	multiplex polymerase chain reaction
NGS	next generation sequencing
PTP	PicoTiterPlate
RNA-Seq	RNA sequencing
SEQC	sequencing quality control
SSNV	somatic single nucleotide variation
WGA	whole genome amplification
